# Is European Medicines Agency (EMA) sepsis criteria accurate for neonatal sepsis diagnosis or do we need new criteria?

**DOI:** 10.1371/journal.pone.0218002

**Published:** 2019-06-06

**Authors:** Funda Tuzun, Hasan Ozkan, Merih Cetinkaya, Ebru Yucesoy, Ozge Kurum, Burcu Cebeci, Ertan Cakmak, Aydan Ozkutuk, Pembe Keskinoglu, Bora Baysal, Abdullah Kumral, Nuray Duman

**Affiliations:** 1 Department of Pediatrics, Division of Neonatology, Dokuz Eylul University Faculty of Medicine, Izmir, Turkey; 2 Department of Pediatrics, Division of Neonatology, Kanuni Sultan Süleyman Training and Research Hospital, Istanbul, Turkey; 3 Department of Pediatrics, Division of Neonatology, Urfa Maternity and Children Hospital, Urfa, Turkey; 4 Department of Clinical Microbiology, Dokuz Eylul University Faculty of Medicine, Izmir, Turkey; 5 Department of Biostatistics and Bioinformatics, Dokuz Eylul University Faculty of Medicine, Izmir, Turkey; All India Institute of Medical Sciences, Bhopal, INDIA

## Abstract

**Background:**

Currently, there is a lack of clear definition for neonatal sepsis. The Pediatric Committee of the European Medicines Agency (EMA) developed consensus criteria to ensure a standardization for neonatal sepsis definition. However, there is no evidence supporting the accuracy of the EMA sepsis criteria in neonatal sepsis diagnosis. The main objective of this study was to evaluate the diagnostic accuracy of EMA sepsis criteria for proven neonatal sepsis.

**Methods:**

A multicenter prospective cohort study was conducted from October 2015 to November 2018. Infants with a gestational age over 34^th^ weeks, diagnosed with clinical sepsis and received antibiotics according to the EMA criteria or experienced neonatologists’ opinion were included. Blood culture or multiplex real time-PCR or 16S-rRNA positive infants were accepted as “proven sepsis”. The predictive performance of EMA criteria for proven sepsis was evaluated by sensitivity, specificity, accuracy, and area under the curve measures of receiver operator characteristic curves. Data-mining methods were used for further analysis.

**Results:**

Among the 245 included infants, the EMA criteria were positive in 97 infants (39.6%), while proven sepsis was diagnosed in 113 infants (46.1%). The sensitivity, specificity, and accuracy of the EMA criteria for proven sepsis were 44.2% (95%CI: 34.9–53.9), 64.4% (95%CI: 55.6–72.5), 55.1% (95%CI: 46.6–59.4) respectively. None of the clinical and laboratory parameters had sufficient performance individually in terms of sensitivity, specificity and accuracy measures. The diagnostic performance was similar when different clinical findings were added to the EMA sepsis criteria or assessment of the score was interpreted in different ways.

**Conclusions:**

Results highlighted that clinician opinion and standard laboratory tests are limited in the neonatal sepsis diagnosis. The EMA criteria also did not efficiently meet the diagnostic accuracy measures for neonatal sepsis. A predictive sepsis definition and rapid bedside point-of care tests are urgently needed.

## Introduction

Neonatal sepsis remains a leading cause of mortality and morbidity although considerable advances have been made in the area of neonatology. Early diagnosis of sepsis is challenging because of the absence of specific symptoms and suboptimal diagnostic value of laboratory tests, which leads to a very low clinical suspicion index and high rates of empiric antimicrobial treatment [1, 2]. An accepted definition of sepsis in neonates is lacking, and a consensus definition for neonatal sepsis is urgently needed. In 2005, the International Pediatric Sepsis Consensus Conference defined sepsis as SIRS in presence of suspicious or definite infection in infants aged above 37 weeks. However, the diagnostic accuracy of certain parameters included in the criteria of SIRS such as white blood cell count and body temperature are insufficient in neonates [3].

In 2010, The Pediatric Committee (PDCO) of the European Medicines Agency (EMA) developed “Expert Meeting on Neonatal and Pediatric Sepsis Consensus 2010 Criteria” and recommended these criteria for standardization of neonatal sepsis definition. According to the report on the expert meeting on neonatal and pediatric sepsis of EMA, neonatal sepsis can be defined by the presence of at least two clinical symptoms and at least two laboratory signs in the presence of or as a result of suspected or proven infection (positive culture, microscopy or polymerase chain reaction) [4]. However, there is no evidence related to the validity and reliability of the EMA sepsis criteria in predicting proven neonatal sepsis. Furthermore, there is also a lack of evidence about the diagnostic value of findings that constitute the EMA sepsis criteria.

The primary objective of this study is to evaluate the diagnostic accuracy of the ‘Expert Meeting on Neonatal and Pediatric Sepsis Consensus 2010 Criteria” in proven neonatal sepsis. In addition, we analyzed the diagnostic value of each clinical sign whether or not included in the EMA sepsis criteria in predicting proven sepsis and tried to establish a modified EMA criteria using additional clinical findings (expanded EMA).

## Patients and methods

### Patients and settings

This study was conducted as a multicenter prospective methodological study, from October 2015 to November 2018. The participating centers were Neonatal Intensive Care Units of Dokuz Eylul University Hospital, Kanuni Sultan Süleyman Training and Research Hospital and Urfa Maternity and Children Hospital. These units are reference centers representing different geographical regions of the country. Ethics approval was obtained from Dokuz Eylul University Faculty of Medicine Ethics Committee.

Term or late preterm newborns over 34^th^ gestational weeks, suspected with sepsis according to the EMA criteria or experienced neonatologists’ opinion and who were to start empirical antibiotic therapy, were eligible. Written informed contents were obtained from the newborns' parents before participation.

The study exclusion criteria were specified as presence of i. major congenital anomaly, confirmed intrauterine infection or metabolic diseases, ii. chorioamnionitis or prolonged premature rupture of the membranes exceeding 18 hours (PPROM), or maternal antibiotic treatment during the last week of pregnancy, iii. antibiotic exposure of the baby within the week before the diagnosis of clinical sepsis, and iv. lack of parental consent.

### Evaluation of clinical sepsis and EMA sepsis criteria

Detailed physical examination was performed in all patients. Presence of at least two clinical and two laboratory signs among the EMA criteria, was considered positive [4]. Besides the clinical findings included in the EMA criteria, 18 additional clinical findings were assessed (jaundice, other respiratory findings, cellulitis, pustule, abscess, ecthyma, erythema multiform, purpura, paleness, hepatomegaly, splenomegaly, diarrhea, melena, hematochezia, tremor, jitteriness, convulsion, tense fontanelle). A modified “expanded EMA” sepsis criteria was established by adding these supplementary clinical findings in to the related categories of original EMA criteria.

Neonatal sepsis was classified as early-onset neonatal sepsis (EONS, <72 h) and late-onset neonatal sepsis (LONS, > 72h) according to the age at the time of onset of the sepsis episode [5].

### Septic work-up

Before treatment, routine sepsis workup was performed in all neonates who were suspected of clinical sepsis and the laboratory parameters included in the EMA criteria were evaluated in all patients (complete blood count, C-reactive protein, blood gases, blood glucose, and immature/total neutrophil ratio with peripheral smear). At least 1 mL blood was taken for blood culture (BD BACTEC Peds Plus/F Culture vials, Becton, Dickinson and Company, USA) and the samples were processed according the Clinical and Laboratory Standards Institute (CLSI) guideline [6]. According to the attending neonatologists’ decision, other body fluid samples (i.e. urine, cerebrospinal fluid, tracheal aspirate) were evaluated and additional studies (i.e. metabolic tests, radiological imaging) were performed for differential diagnosis.

### Real-time PCR based identification

Positive blood culture is still gold standard for the diagnosis of sepsis, however, the microorganism detection rate is low (25–40%). Therefore, polymerase chain reaction (PCR) based tests (The Roche LightCycler SeptiFast MGRADE PCR, and 16S-rRNA PCR) were used to increase the microorganism detection rate. A positive blood culture or positive PCR-based test was considered reference standard for proven sepsis. The Roche LightCycler SeptiFast MGRADE is a commercially available certified multiplex real-time PCR system with simultaneous analysis of 20 different pathogens which are commonly retrieved pathogens in neonatal units [7]. This system using a modified DNA extraction protocol showed acceptable results for rapid detection of neonatal sepsis in addition to conventional blood culture. For culture-positive neonatal sepsis, it has a sensitivity of 80–90% and a specificity of 72–80% (95%CI 67.0–79.0%) [7, 8].

At the time of clinical sepsis diagnosis, an additional 2mL sterile blood sample was obtained before antibiotic treatment and 1.5mL was used for nucleic acid extraction. The nucleic acid extracts were stored at -20C. The following principal steps were performed according to the published protocols: (i) extraction and purification of DNA from whole blood; (ii) Real-time PCR amplification of target DNA in three parallel reactions (Gram positive, Gram negative, fungi) and subsequent detection of PCR products by specific hybridization probes, and (iii) automated identification of species and controls. A predefined semi quantitative analytical CT cut-off value at 20 cycles was applied for CoNS and Streptococcus spp. in order to reduce cross-contamination [7, 9]. Broad range 16S rRNA was also performed to increase the sensitivity of multiplex PCR and to ensure control of PCR based methods [10] [11].

Blood samples negative for bacterial or fungal microorganisms were further evaluated with PCR using Roche LightMix Modular Enterovirus and Roche LightMix Adenovirus tests using the LightCycler 480 System.

### Statistical analysis

In univariate analysis, the categorical variables were compared using the McNemar’s chi-square test for dependent groups and the chi-square analysis for independent groups. The variables which had a p value of <0.100 were also examined with logistic regression analysis.

The sensitivity, specificity, positive predictive value (PPV), negative predictive value (NPV), and accuracy of the EMA criteria for proven sepsis were calculated with standard two-by-two tables, through MedCalc Statistical Software version 16.4.3 (MedCalc Software bvba, Ostend, Belgium; https://www.medcalc.org; 2016)[12]. The predictive value of EMA criteria, expanded EMA criteria and different combinations of clinical and laboratory findings were tested using the classical multivariate logistic regression analysis. The test score performances (area under the curve [AUC], sensitivity [recall], positive predictive value [precision], and ROC curves were evaluated by applying the data mining methods including the support vector machine (SVM), decision tree, random forest, naive-bayes, neural network and logistic regression algorithms.

Minimum sample size required was calculated as 198 patients using one ROC curve power analysis, considering the proven sepsis prevalence as 0.40, for AUC: 0.70, α = 0.05 beta, β = 0.20 [13]. The sample size needed for the targeted main result was not affected by the “missing data”, because the patients who lack of index test (EMA criteria) and reference standard tests (blood culture, multiplex real- time PCR and 16S-rRNA PCR) did not meet the study criteria.

In all statistical comparisons, a p value of <0.05 was considered statistically significant. The SPSS 22.0 package program and the free software Orange 3.18.0 directed to data mining analysis were used in the analyses.

## Results

**Fig 1:**
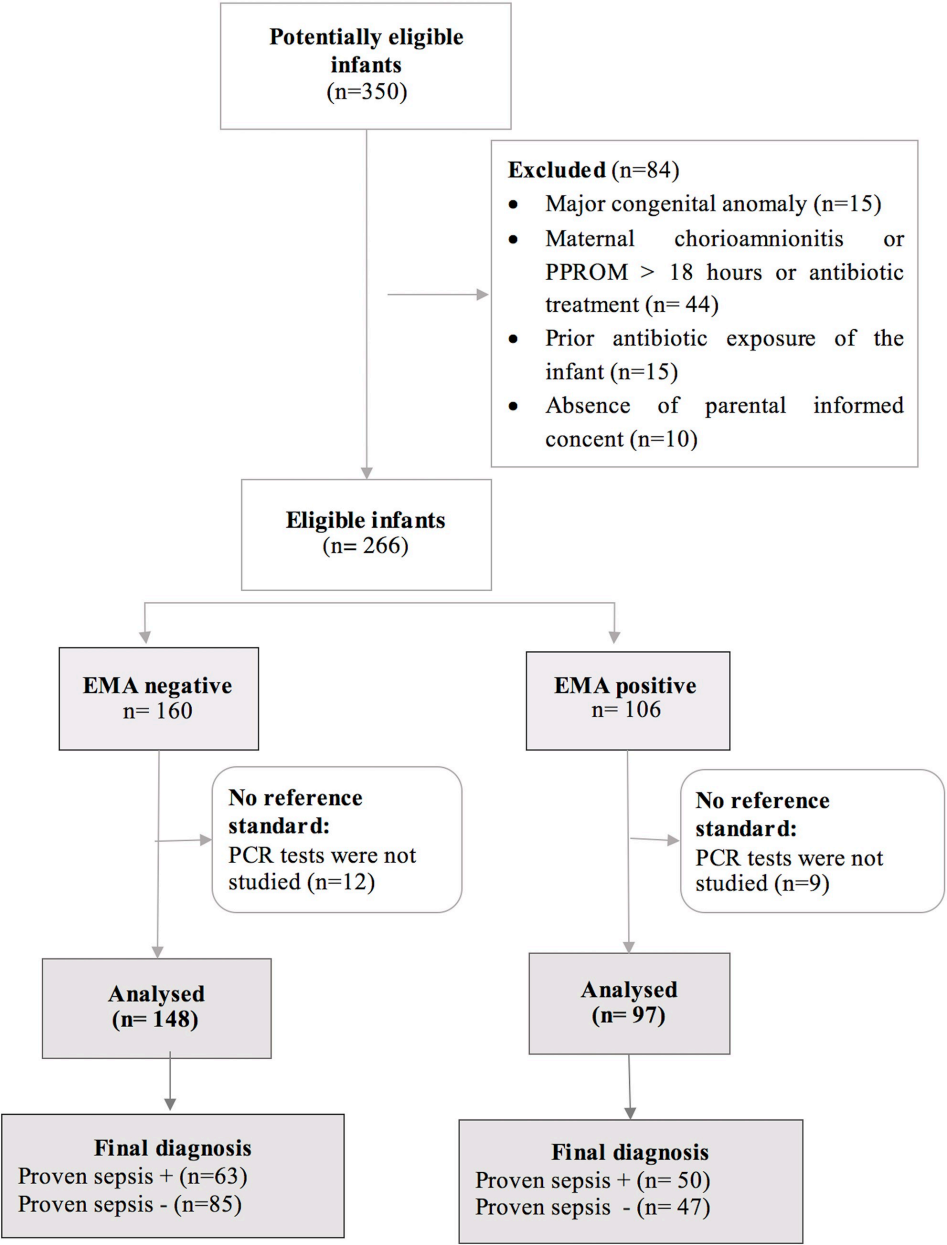
Flow diagram of the patients. 245 infants were included to the study among 350 potentially eligible infants.

During the three years study period, a total of 245 babies fully met the study criteria (Fig 1). The basic demographic and clinical data of the study group are shown in Table 1.

**Table 1:** Characteristics of the study population.

**Perinatal characteristics**	**N = 245**
**Gestational age in weeks, mean ±SD ^a^**	38.4 ±1.5
**< 37 gestational weeks**	20 (8.2%)
**≥ 37 gestational weeks**	225 (91.8%)
**Birth weight in gr, mean ±SD**	3336.1 ± 518.1
**Type of birth, cesarean section n (%)**	101 (41.2%)
**Female gender, n (%)**	97 (39.6%)
**1^st^ min Apgar score, median (IQR)^b^**	8 (7–9)
**5^th^ min Apgar score, median (IQR)^b^**	9 (9–10)
**Age at onset of suspected infection (day), median (IQR) ^b^**	1.25 (0.41–2.45)
**EONS (≤72 hours) n (%)**	195 (79.6%)
**LONS (>72 hours) n (%)**	50 (20.4%)
**Clinical follow up**	
**Duration of antibiotic treatment in days, mean ±SD**	8.7 ±3.0
**Mechanical ventilation requirement, n (%)**	91 (37.1%)
**Inotrope treatment requirement, n (%)**	4 (1.6%)
**Duration of hospitalization in days, mean ±SD**	12.7 ±10.8
**Mortality during hospitalization, n (%)**	2 (0.8%)

^**a**^ SD: standard deviation

^**b**^ IQR: inter-quartile range

The number of patients who met the EMA criteria according to the standard definition (2 clinical and 2 laboratory findings) was 97 (39.6%). Among the all evaluated clinical parameters, included or not in the EMA criteria, absence of suckling (n = 198), feeding intolerance (n = 168) and jaundice (n = 88) were the most common ones. The most common clinical findings, that are not included in the EMA criteria were jaundice (n = 88), jitteriness (n = 16) and additional respiratory symptoms (n = 12).

Blood culture was positive in 31 patients (11.4%), while “SeptiFast Multiplex PCR” or “16S rRNA” tests were positive in 103 patients (42%). Considering the results of blood culture and PCR based tests together, 113 patients (46.1%) diagnosed as proven sepsis. Species of coagulase-negative staphylococci (CoNS) were the most common microorganisms detected by both tests (blood culture and The Roche SeptiFast MGRADE PCR) and followed by Klebsiella species. *(pneumonia/oxytoca)*. The other detected species included Group B streptococcus, Streptococcus pneumonia, Staphylococcus aureus, Escherichia coli, Pseudomonas aeruginosa, Acinetobacter baumannii, Enterobacter cloacae, Serratia marcescens and Candida albicans. Viral PCR tests performed for bacteria negative patients, were negative.

All of the evaluated clinical and laboratory parameters, whether or not included in the EMA sepsis criteria, were evaluated in terms of predicting proven sepsis, through univariate analysis and diagnostic accuracy measures. None of these parameters had sufficient performance individually in terms of sensitivity, specificity and accuracy measures. The only significant parameter was body temperature abnormality; its specificity appeared well, but its sensitivity and accuracy values were low (Table 2). Backward stepwise logistic regression analysis including all of the EMA criteria, demonstrated that only body temperature abnormality remained in the final model after elimination. In this model, AUC was 0.614 (95% CI: 0.570–0.715, p = 0.002).

**Table 2:** Diagnostic accuracy measures of the clinical and laboratory findings included in the EMA sepsis criteria at the time of sepsis suspicion^a^.

	n /n’ (%) ^b^	OR (95% GA)	p	Sensitivity %	Specificity %	Accuracy %
**Body temperature abnormality ^c^**	23/30 (76.7)	4.5 (1.8–11.0)	<0.001	20.35	94.70	60.41
**Bradycardia, tachycardia or rhythm instability ^d^**	4/5 (80.0)	4.8 (0.5–43)	0.101	3.54	95.85	55.10
**Oliguria ^e^**	2/2 (100)	-	0.212	1.77	100	54.69
**Hypotension ^f^**	2/3 (66.7)	2.4 (0.2–26.4)	0.596	1.77	99.24	54.29
**Mottled skin**	46/91 (50.5)	1.3 (0.8–2.2)	0.285	40.71	65.91	54.29
**Impaired peripheral perfusion**	46/92 (50.0)	1.3 (0.8–2.1)	0.345	40.71	65.15	53.88
**Petechial rash**	2/3 (66.7)	2.3 (0.2–26.4)	0.596	1.77	99.24	54.29
**Sclerema**	0 (0)	**-**	**-**	**-**	**-**	**-**
**Apnea**	4/8 (50.0)	1.2 (0.3–4.8)	0.823	3.54	96.97	53.88
**Tachypnea**	39/80 (48.8)	1.2 (0.7–2.0)	0.566	34.51	68.94	53.06
**increased oxygen requirements**	47/93 (50.5)	1.3 (0.8–2.2)	0.278	41.59	65.15	54.29
**Requirement for ventilation support**	42/88 (47.7)	1.1 (0.6–1.9)	0.706	37.17	65.15	52.24
**Feeding intolerance**	77/168 (45.8)	1.0 (0.8–1.4)	0.893	68.14	31.06	48.16
**Poor sucking**	91/198 (46.0)	1.1 (0.7–1.4)	0.916	80.53	18.94	47.35
**Abdominal distention**	4/6 (66.7)	2.4 (0.4–13.3)	0.419	3.54	98.48	54.69
**Irritability**	12/27 (44.4)	1.1 (0.7–1.6)	0.853	10.62	88.64	52.65
**Lethargy**	9/14 (64.3)	2.4 (0.2–26.4)	0.596	7.96	96.21	55.**51**
**Hypotonia**	19/34 (55.9)	1.6 (0.8–3.2)	0.219	16.81	88.64	55.51
**WBC count abnormality ^g^**	33/67 (49.3)	1.2 (0.7–2)	0.546	29.20	74.24	53.47
**(I/T)> 0.20 ^h^**	55/117 (47.0)	1.1 (0.6–1.8)	0.790	48.67	53.03	51.02
**Thrombocytopenia ^i^**	8/13 (61.5)	1.9 (0.6–6.1)	0.252	7.08	96.21	55.10
**C reactive protein> 15 mg/L**	52/103 (50.5)	1.4 (0.8–2.2)	0.243	46.02	61.36	54.29
**Glucose intolerance ^j^**	8/17 (47.1)	1.0 (0.4–2.8)	0.936	7.08	93.18	53.47
**Base excess (BE) <-10 mEq/L**	2/6 (33.3)	1.37 (0.4–4.3)	0.541	1.83	96.92	53.56
**Serum lactate >2 mMol/L**	68/149 (45.6)	1.0 (0.6–1.7)	0.99	62.39	37.69	48.95

**a.** Total sample size = 245

**b. n** represents the frequency of proven sepsis among the cases presenting with certain finding (**n’**)

**c.** Core temperature greater than 38,5°C or less than 36°C and/or temperature instability

**d.** Bradycardia (mean HR less than the 10th percentile for age in the absence of external vagal stimulus, beta blockers or congenital heart disease OR otherwise unexplained persistent depression over a 0.5 h time period) OR tachycardia (mean HR greater than 2 SD above normal for age in the absence of external stimulus, chronic drugs and painful stimuli OR otherwise unexplained persistent elevation over a 0,5 h to 4 h time period) and/or rhythm instability

**e.** Reduced urinary output (less than 1 mL/kg/h)

**f.** Hypotension (mean arterial pressure less than the 5th percentile for age)

**g.** White blood cells (WBC) count: <4000x x109 cells/L or 20000 x109 cells/L

**h.** Immature to total neutrophil ratio (I/T) greater than 0.2

**i.** Platelet count <100000 x109 cells/L

**j.** Glucose intolerance confirmed at least 2 times: hyperglycemia (blood glucose >180 mg/dL or 10 mMol/L) OR hypoglycemia (blood glucose < 45 mg/dL or 2.5 mMol/L) when receiving age specific normal range glucose amounts

**Fig 2:**
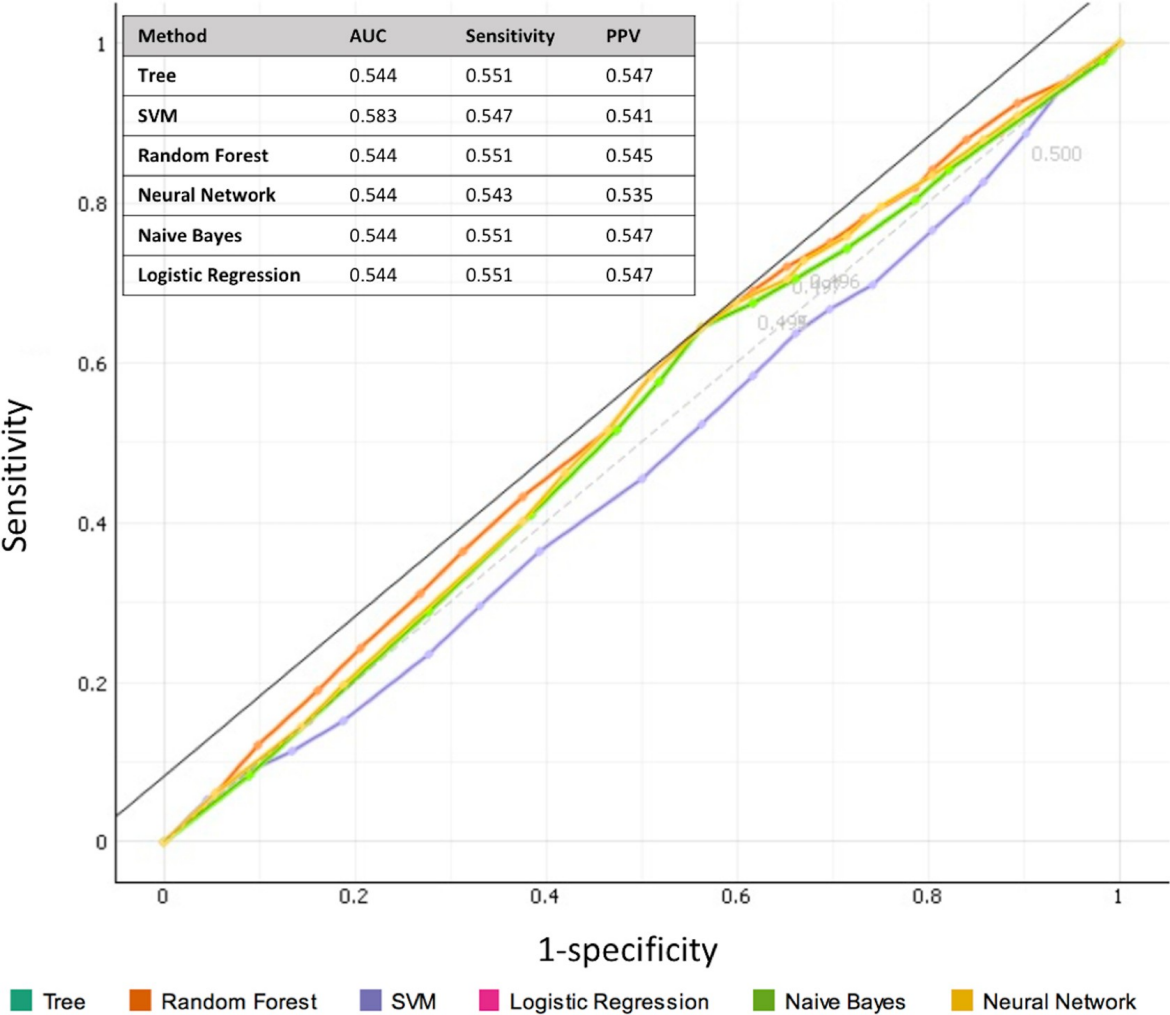
Evaluation of diagnostic value of the EMA sepsis criteria using data mining methods. Area under the curve [AUC], sensitivity, positive predictive value [PPV] scores and ROC curves evaluated by decision tree, support vector machine (SVM), random forest, naive-bayes, neural network, and logistic regression algorithms are shown.

The sensitivity and specificity of the EMA sepsis criteria in predicting proven sepsis were 44.2% and 64.4%, respectively (Table 3). Evaluation of diagnostic value of the EMA sepsis criteria using data mining methods did not change the results dramatically, as the AUC was 0.583 at most and the sensitivity and PPV values were insufficient (Fig 2).

**Table 3:** Diagnostic accuracy measures^a^ of EMA sepsis criteria and its modifications: In total sample and subgroups of EONS and LONS^b^.

	Criteria	Sensitivity (95% CI)	Specificity (95% CI)	PPV (95% CI)	NPV (95% CI)	Accuracy (95% CI)
**Total sample (n = 245)**	**EMA**	44.2 (34.9–53.9)	64.4 (55.6–72.5)	55.6 (43.8–59.2)	57.4 (52.3–62.4)	55.1 (46.6–59.4)
	**Expanded EMA**	49.6 (40.0–59.1)	56.1 (47.2–64.7)	49.1 (42.5–55.8)	56.5 (50.6–62.2)	53.1 (46.6–59.4)
	**EMA (3 clinical +2 laboratory)**	34.5 (25-8-44.0)	73.5 (65.1–80.8)	52.7 (43.2–61.9)	56.7 (52.5–60.8)	55.5 (49.0–61.84)
	**EMA (2 clinical +3 laboratory)**	27.4 (19.5–36.6)	72.7 (64.3–80.1)	46.3 (36.4–56.5)	53.9 (50.1–57.7)	51.8 (45.4–58.2)
**EONS (n = 195)**	**EMA**	43.9 (33.6–54.8)	61.5 (51.5–70.9)	50.0 (41.7–58.3)	55.6 (49.8–61.4)	53.3 (46.1–60.5)
	**Expanded EMA**	49.4 (38.8–60.1)	54.8 (44.7–64.6)	48.9 (41.6–56.3)	55.3 (48.7–61.8)	52.3 (45.0–59.5)
**LONS (n = 50)**	**EMA**	45.4 (24.4–67.8)	75.0 (55.1–89.3)	58.8 (39.4–75.9)	63.6 (53.0–73.0)	62.0 (47.2–75.6)
	**Expanded EMA**	50.0 (28.2–71.8)	60.7 (40.6–78.5)	50.0 (34.9–65.1)	60.7 (48.1–72.1)	56.0 (41.2–70.0)

^**a**^ The sensitivity, specificity, positive predictive value (PPV), negative predictive value (NPV), and accuracy measures of the criteria for proven sepsis were calculated with standard two-by-two tables through MedCalc statistical software.

^**b**^ EONS: Early onset neonatal sepsis; LONS: Late onset neonatal sepsis

To develop a more predictive score, the other clinical parameters which were not included in the EMA sepsis criteria were added to the relevant categories and defined as “expanded EMA”. Expanded EMA criteria was also accepted positive by the presence of at least 2 clinical and 2 laboratory findings. In this way, the number of EMA positive patients increased to 114 (46.5%). The sensitivity and specificity measures of the expanded EMA criteria for predicting proven sepsis were 49.6% and 56.1% respectively (Table 3). In addition, when the EMA criteria was evaluated with different cut-off values for laboratory parameters, diagnostic value did not change. Diagnostic performance of EMA criteria was decreased if presence of 3 clinical and 2 laboratory findings or 2 clinical and 3 laboratory findings were accepted as positive (Table 3).

Eighty percent of the patients (n = 195) were diagnosed as EONS and the results were thought to reflect the characteristics of EONS to a large extent. Therefore, the diagnostic accuracy measures of the EMA and expanded EMA criteria were evaluated separately in EONS and LONS subgroups. In both groups, their performances were insufficient, although a slight increment of sensitivity was detected in LONS subgroup (Table 3). Nevertheless, sample size of LONS subgroup was underpowered.

## Discussion

Results of the present study revealed that EMA sepsis criteria did not efficiently meet the diagnostic accuracy measures for proven neonatal sepsis. The diagnostic performance was far below the ideal levels, when different clinical findings were added to the EMA sepsis criteria or assessment of the score was interpreted in different ways. These data emphasize the restricted accuracy of initial clinician opinion and standard laboratory tests to detect bacterial infection, especially for EONS. Results highly support the invitation of Wynn and Polin for an urgent consensus for the diagnosis and definition of neonatal sepsis [14, 15].

Currently, clinical sepsis diagnosis reflects to the clinician’s suspicion in presence of nonspecific clinical and laboratory findings. EONS is generally manifested with respiratory distress, apnea, lethargy or irritability, temperature instability, and feeding difficulties. These symptoms are nonspecific, because many non-infected newborns may show similar symptoms. During the first days of life, different organ systems adapt to extra-uterine life dynamically. A single-point, clinical assessment to diagnose EONS therefore seems impossible [16]. Although 80–100% of the infants with culture positive EONS show clinical signs consistent with sepsis in the first 48 hours after birth, those signs are not specific. EONS was diagnosed in only 2.7–5.6% of the infants who had such clinical findings. This means that 18–38 babies would receive unnecessary antibiotic treatment to treat one newborn with bacterial sepsis [17].

The positive predictive values of hematological parameters and inflammatory markers used in the diagnosis of neonatal sepsis are low and serial measurements are required [18]. Insufficient diagnostic performance of these tests may be related to perinatal inflammatory reactions triggered by factors during delivery or in the early postnatal period [16]. It is unavoidable that the hematological parameters and inflammatory markers or scoring systems constituted with combination of these, will lead to unnecessary antibiotic usage in babies with suspicious sepsis.

In this study, none of the evaluated clinical or laboratory signs showed an adequate diagnostic performance for predicting proven sepsis. Therefore, an advanced scoring system composed of predictive parameters for proven sepsis could not be developed using classical logistic regression analysis and data mining methods. Expanded EMA criteria showed a slight improvement in sensitivity, however its diagnostic accuracy was still insufficient. The laboratory findings included in the EMA criteria are also nonspecific and the cut-off values are unadjusted for postnatal age [4, 16]. Therefore, different cut-off values were tested in our study, however this effort did not lead to a significant increase in the diagnostic performance of the EMA criteria.

There has been one observational study exists in the literature evaluating the diagnostic accuracy of the EMA criteria in preterm babies [19]. In this study, the PPV of the EMA criteria was 61% for blood culture positive LONS and the EMA criteria were interpreted as acceptable. In our study, the sensitivity and specificity measures were suboptimal even for late-onset sepsis.

A positive blood culture is still the “gold standard” for proven neonatal sepsis in presence of clinical suspicion. However, conventional blood culture has some limitations including the requirement of a long-time period for the results, low sensitivity due to small blood sampling volumes, low-concentration bacteremia in neonates and suppression of bacterial growth by preceding antibiotic treatment. [18, 20] Even today, the highest detection rate of blood culture is about 40% [21]. These limitations can be overcome with PCR-based rapid diagnostic tests. In recent years, 16S-rRNA and multiplex PCR tests have been intensively evaluated for timely and accurate diagnosis of sepsis [11, 22, 23]. Especially, prompt results in 6 hours is a very important advantage of PCR in critically ill patients. Recent studies have shown that The Roche SeptiFast MGRADE PCR shows acceptable results for rapid detection of neonatal sepsis in addition to conventional blood culture, though certain limitations exist [7, 8].

This is the first study evaluating the diagnostic accuracy of the EMA criteria in proven neonatal sepsis accompanied with bacteriological culture and current PCR-based methods. The strong aspects of this study include a prospective-multicenter design of the study and utilization of current PCR based tests for proven sepsis diagnosis. Use of PCR-based molecular methods increases diagnostic sensitivity of laboratory tests considering the low rate of blood culture positivity [7, 8, 22–24]. Another strength of this study was performing data mining methods for analysis, which is a new technique that develop the artificial intelligence and database technique in recent years and basically enables multidimensional evaluation of the complex interactions.

The use of PCR techniques may bring some disadvantages as well as advantages. One important limitation of PCR is differentiation of potential contamination from true positive cases, particularly for accurate CoNS detection [8, 23]. To reduce cross-contamination in this study, a pre-defined semi quantitative analytical CT cut-off value at 20 cycles was applied for CoNS and Streptococcus spp.[9]. Another limitation of molecular-based detection methods is that they can’t discriminate living vs. dead bacteria in antibiotic exposed patients and higher rates of false positive results are inevitable. On the other hand, it provides important information about antibiotics-suppressed pathogens. To avoid confusion, the patients who had previous antibiotic exposure were not included to the study.

Another limitation that could lead to bias was the predominance of EONS in the study sample and the mortality and morbidity rates were much lower compared to the rates exist in the literature [25]. It is unknown how the results could be influenced, if more critically ill patients were included in the study. However, the study has a prospective multicenter design and completely reflects the common clinical practice.

## Conclusions

Neonatal sepsis is a dynamic, complex and heterogeneous physio-pathological process. The clinical findings are ambiguous and diagnostic performance of current inflammatory biomarkers show inconsistency depending on the process. Therefore, critical problems arise in the prompt and accurate diagnosis of neonatal sepsis with use of static definitions and assessments. Hopefully in the near future, an integration of clinical signs, laboratory and sophisticated molecular tests including multi-omics technologies and microarray chips will enable for timely and accurate diagnosis of neonatal sepsis.

## Supporting information

S1 Data Raw data of the patients. (SAV)
